# Six rounds of annual praziquantel treatment during a national helminth control program significantly reduced schistosome infection and morbidity levels in a cohort of schoolchildren in Zimbabwe

**DOI:** 10.1371/journal.pntd.0008388

**Published:** 2020-06-22

**Authors:** Takafira Mduluza, Caitlin Jones, Derick N. M. Osakunor, Rivka Lim, Julius K. Kuebel, Isaac Phiri, Portia Manangazira, Paradzayi Tagwireyi, Francisca Mutapi

**Affiliations:** 1 Department of Biochemistry, University of Zimbabwe, Mount Pleasant, Harare, Zimbabwe; 2 TIBA Zimbabwe, NIHR Global Health Research Unit Tackling Infections to Benefit Africa (TIBA) at the University of Edinburgh, Edinburgh, United Kingdom; 3 Centre for Infection, Immunity & Evolution and Institute of Immunology & Infection Research, University of Edinburgh, Ashworth Laboratories, King's Buildings, Edinburgh, United Kingdom; 4 NIHR Global Health Research Unit Tackling Infections to Benefit Africa (TIBA) at the University of Edinburgh, Ashworth Laboratories, King's Buildings, Edinburgh, United Kingdom; 5 Epidemiology & Disease Control, Ministry of Health and Child Care, Harare, Zimbabwe; 6 Department of Geography and Environmental Science, Geo-information and Earth Observation Centre, University of Zimbabwe, Mount Pleasant, Harare, Zimbabwe; Centers for Disease Control and Prevention, UNITED STATES

## Abstract

**Background:**

The World Health Organization recommends that schistosomiasis be treated through Mass Drug Administration (MDA). In line with this recommendation, Zimbabwe commenced a national helminth control program in 2012 targeting schoolchildren throughout the country for 6 years. This study, part of a larger investigation of the impact of helminth treatment on the overall health of the children, determined the effect of annual praziquantel treatment on schistosome infection and morbidity in a cohort of children during Zimbabwe’s 6-year national helminth control program.

**Methodology/Principal findings:**

A school-based longitudinal study was carried out in 35 sentinel sites across Zimbabwe from September 2012 to November 2017. The sentinel sites were selected following a countrywide survey conducted in 280 primary schools. *Schistosoma haematobium* was diagnosed using the urine filtration technique. *Schistosoma mansoni* was diagnosed using both the Kato-Katz and formol-ether concentration techniques. *S*. *haematobium* morbidity was determined through detection of macro and microhaematuria. A cohort of children aged 6–15 years old was surveyed annually before MDA and 6 weeks post treatment. Maximum treatment coverage reached 90% over the 6 rounds of MDA. At baseline *S*. *haematobium* infection prevalence and intensity were 31.7% (95% CI = 31.1–32.2) and 28.75 eggs/10ml urine (SEM = 0.81) respectively, while *S*. *mansoni* prevalence and intensity were 4.6% (95% CI = 4.4–4.8) and 0.28 eggs/25mg (SEM = 0.02). Prior to the 6^th^ round of MDA, *S*. *haematobium* infection prevalence had reduced to 1.56% (p<0.001) and infection intensity to 0.07 (SEM 0.02). Six weeks later after the 6^th^ MDA, both were 0. Similarly the prevalence of *S*. *haematobium* morbidity as indicated by haematuria also fell significantly from 32.3% (95% CI = 29.9–34.6) to 0% (p< 0.0001) prior to the final MDA. For *S*.*mansoni*, both prevalence and intensity had decreased to 0 prior to the 6^th^ MDA. After 6 rounds of annual MDA, prevalence and intensity of both schistosome species decreased significantly to 0% (p< 0.0001).

**Conclusion:**

Zimbabwe’s helminth control program significantly reduced schistosome infection intensity and prevalence and urogenital schistosomiasis morbidity prevalence in a cohort of school-aged children, moving the schistosome prevalence in the children from moderate to low by WHO classification. These findings will inform the design of the country’s next stage interventions for helminth control and eventual elimination.

## Author summary

Following a national helminth prevalence survey conducted in 2010/2011, Zimbabwe launched a national schistosomiasis and intestinal worm control program in 2012 targeting school children aged 6–15 years who carry the highest infection burden. The control strategy as advocated by the World Health Assembly in 2012 was adopted to suit the country context. We conducted a study in a cohort of children to assess the effect of praziquantel treatment on schistosome infection intensity and prevalence as well as on urogenital schistosomiasis morbidity prevalence after every annual MDA for 6 years. Maximum treatment coverage in a cohort of children reached 90% over the 6 rounds of annual treatment. Annual surveys in sentinel sites throughout the country showed that schistosome infection prevalence and intensity as well as the marker of urogenital schistosomiasis, haematuria decreased significantly after each round of treatment from the pre-treatment levels in the same year. Most notably, both urogenital schistosomiasis infection and morbidity prevalence reduced from 32% at baseline in 2012 to 1.56% before the 6^th^ MDA in 2017, and infection intensity reduced from 28.75 eggs/10ml urine to a mean of 0.07eggs/10ml urine prior to the 6^th^ MDA. Both infection prevalence and intensity fell to 0 after the 6^th^ MDA. Intestinal schistosomiasis prevalence and intensity reduced from 4.6% and 0.28 eggs/25mg of stool to 0% and 0 eggs, respectively. Thus, the control program significantly reduced schistosome infection and morbidity in this cohort of schoolchildren.

## Introduction

Zimbabwe is endemic to several of the neglected tropical diseases (NTDs) as listed by the World Health Organization (WHO) [1]. Of these NTDs, schistosomiasis is the most prevalent in the country. Both forms of schistosomiasis; urogenital and intestinal, occur in Zimbabwe with the former being more prevalent [2]. From the very first community survey in Zimbabwe, then Rhodesia, in 1932 [3], Zimbabwe has had programs to control schistosomiasis [4]. These programs have targeted workers and schoolchildren using several approaches including treatment of infected people (first with tartar emetic and then praziquantel(PZQ)), improving water and sanitation, snail control and education/awareness campaigns [4]. However, these programs have mostly been focal in space and time [5–8]. In 2012 the WHO set out a roadmap for NTDs, including controlling schistosomiasis morbidity by 2020 and elimination of schistosomiasis as a public health problem and interrupting transmission in various African countries by 2025 [9]. As part of implementing this roadmap, Zimbabwe has been conducting a national helminth control program targeting school children aged 6 to 15 years, whom previous studies in the country have shown to carry the heaviest burden of schistosome infection in Zimbabwe [8, 10–13].

To inform the national control program, we performed a nationwide school-based survey in 2010/2011, mapping *S*. *mansoni*, *S*. *haematobium*, and the soil-transmitted helminth species: *Ascaris lumbricoides*, *Trichuris trichiura* and hookworm [2]. This was done by collecting data at district level, and thereby representative of the corresponding provinces. Data from this study revealed high schistosomiasis prevalence in 55 districts and soil transmitted helminths occurring in 47 districts, with 33 districts being endemic to both types of NTDs. The nationwide combined prevalence of schistosomiasis was 22.7% while that for STH was 5.5% [2]. These levels of infection demanded urgent interventions. Following this, the Ministry of Health formulated a national NTD control policy, an initial 5-year action plan and implemented school-based mass drug administration (MDA) targeting primary and secondary school children across the country [4]. Crucially, Zimbabwe’s control program treated all children annually irrespective of the regional prevalence of infection. This is in contrast to the WHO preventive chemotherapy guidelines that recommend the frequency of treatment based on community/regional infection level, i.e. high risk communities, those with a prevalence of 50% and over, should be treated annually, whereas those at lower risk should be treated every two years (over 10% but less than 50%). In cases where prevalence was less than 10%, then the recommendation is that children should be treated twice within their school career [14] [15].

While data on the coverage of the country’s helminth control program has been documented (see http://espen.afro.who.int/countries/zimbabwe), there had been no indication of the effectiveness of the control program in terms of efficacy of praziquantel treatment on infection intensity and prevalence as well as on levels of urogenital schistosomiasis morbidity as measured by haematuria. This study is part of an investigation on the impact of schistosomiasis treatment with PZQ during the national control program on overall human health including morbidity markers, inflammatory markers, allergic responses and autoreactivity in school children. For this study, we determined egg reduction rates (ERR) and cure rates (CR) in a cohort of school children. This current study focuses on the effect of annual PZQ treatment on schistosome infection intensity and prevalence and urogenital schistosomiasis morbidity in this cohort of school children during Zimbabwe’s national helminth control program and compares infection levels from the start of the MDA to our last survey six weeks after the 6^th^ round of MDA.

## Materials and methods

### Ethical approval and consent

The study received institutional approval from the University of Zimbabwe and Ethical approval from the Medical Research Council of Zimbabwe MRCZ/A/1710. Permission to conduct the study in the provinces was obtained from the Ministry of Health & Child Care. Recruitment into the study was voluntary and parents/guardians gave written parental consent and were free to withdraw the participants at any time with no further obligation.

### Background and national MDA

Between September 2010 and August 2011, a national schistosomiasis and soil transmitted helminths survey was conducted in Zimbabwe by the Ministry of Health and Child Welfare through the Epidemiology and Disease Control Department and National Institute of Health Research with support from the Ministry of Education, the WHO and UNICEF [2].

In keeping with the observation that school children constitute the high-risk age group for schistosomiasis and STH in the community [14], the survey was conducted in school children aged 10–15 years old. The survey was a countrywide cross-sectional survey carried out by sampling 50 children in each of 280 primary schools in all the 68 districts of the country’s total eight provinces. The national survey indicated the different districts in the country which fell in the WHO classification of heavy, moderate and low schistosome endemicity based on the infection levels in the school children [15].

Following this national survey, annual MDAs were carried out in September 2012, October 2013, January 2015, November 2015, November 2016 and November 2017. The third MDA was delayed due to logistical reasons, resulting in the reduced gap between the third and fourth MDA being less than 12 months. The children in the cohort study described here were treated during these MDAs.

### Study design

**Fig 1:**
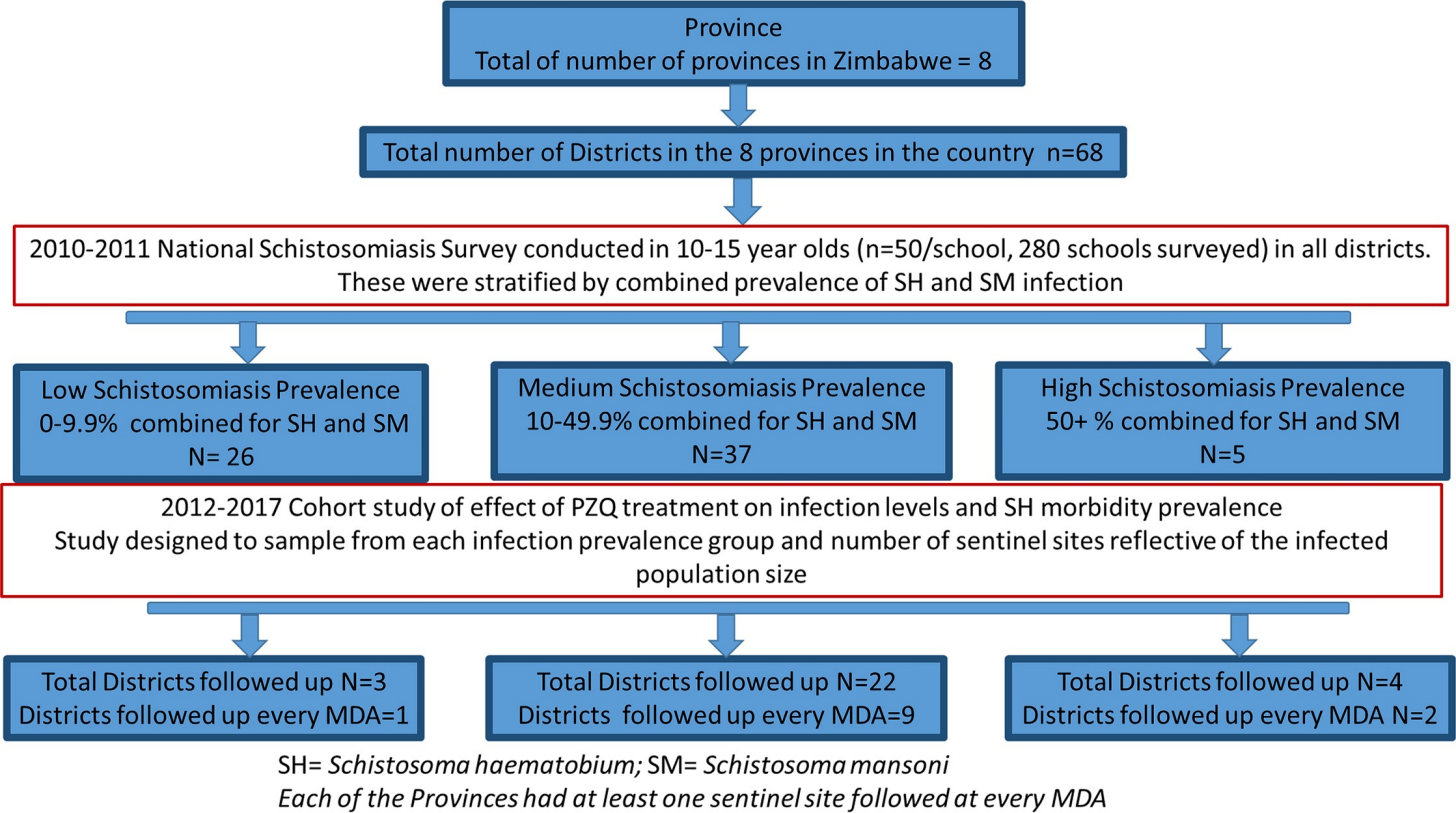
Study design. The schistosome endemicity levels (low, medium, high) were stratified based on combined *S*. *mansoni* and *S*. *haematobium* infection prevalence.

**Fig 2:**
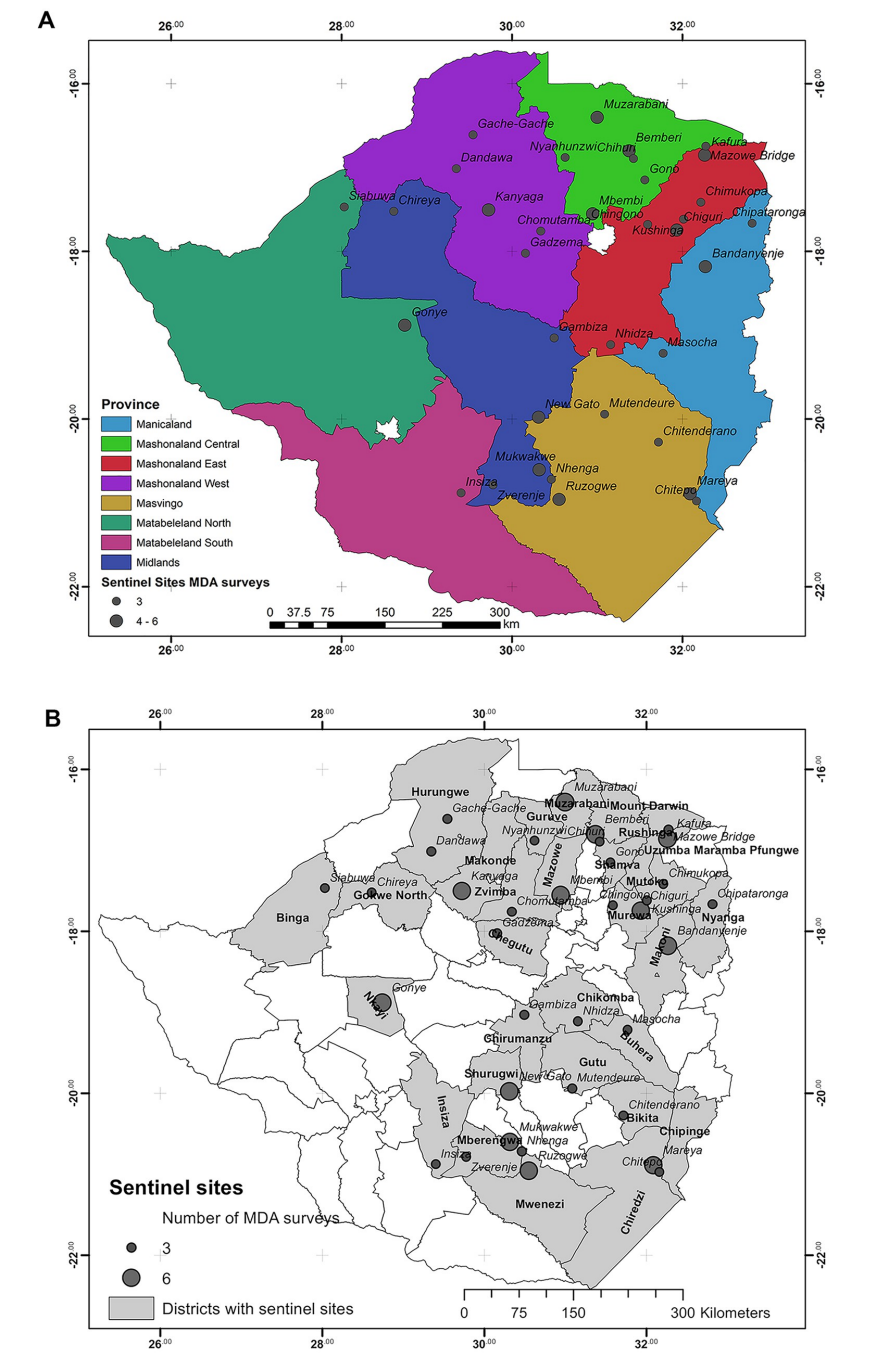
Maps showing sentinel sites at different administration levels. The maps were generated using GIS raw data for the schools using ArcMap 10.1. Sentinel sites are italicized and the number of MDA surveys indicated**. A.** Map showing sentinel sites at province level. **B.** Maps showing sentinel sites at different administration levels. The maps were generated using GIS raw data for the schools using ArcMap 10.1. Map showing sentinel sites at district level.

The study presented here was designed to follow a cohort of primary school children who were targeted by this MDA from September 2012 to November 2017. Sites throughout the country were purposely chosen before the commencement of the MDA program to represent the 3 levels of schistosomiasis infection prevalence as described by WHO i.e. low, moderate and high [14] at both district and province level as shown in **Fig 1**. The sentinel sites were also chosen to represent the baseline prevalence classification of the district (low, medium or high) (see **S1 Fig**). In total 35 schools located in 29 districts and 8 provinces were included in the study. The sample sizes are given in **Table 1** and the map of the 8 provinces is given in **Fig 2A.**

**Table 1:** Summary study sample sizes by MDA.

Province	District	School	Total number of surveys	Number of children surveyed per MDA					
				1	2	3	4	5	6
Manicaland	Bikita	Chitenderano	2	195	-	-	-	-	195
Manicaland	Buhera	Masocha	6	242	-	-	242	-	242
Manicaland	Chipinge	Chitepo	6	250	-	-	250	-	250
Manicaland	Makoni	Bandanyenje	12	242	242	211	242	242	193
Manicaland	Nyanga	Chipataronga	6	398	-	-	398	-	398
Mashonaland Central	Guruve	Nyanhunzi	6	179	-	-	179	-	179
Mashonaland Central	Mazowe	Mbebi	6	186	-	-	186	-	186
Mashonaland Central	Mt Darwin	Bemberi	12	201	206	206	222	196	145
Mashonaland Central	Muzarabani	Muzarabani	12	298	297	298	298	298	213
Mashonaland Central	Rushinga	Mazowe Valley	12	250	250	250	250	250	208
Mashonaland Central	Shamva	Chihuri	6	218	-	-	218	-	218
Mashonaland Central	Shamva	Gono	3	200	-	-	-	-	200
Mashonaland East	Chikomba	Nhidza	6	250	-	-	250	-	250
Mashonaland East	Murewa	Chiguri	12	198	198	198	198	198	175
Mashonaland East	Murewa	Chingono	6	224	-	-	224	-	224
Mashonaland East	Mutoko	Chimukopa	12	250	198	224	224	224	201
Mashonaland East	Mutoko	Kushinga	6	198	-	-	198	-	198
Mashonaland East	UMP	Kafura	12	250	250	250	250	250	226
Mashonaland West	Chegutu	Gadzema	6	267	-	-	267	-	267
Mashonaland West	Hurungwe	Dandawa	6	206	-	-	206	-	206
Mashonaland West	Hurungwe	Gache-gache	6	233	-	-	233	-	233
Mashonaland West	Makonde	Kanyanga	6	250	-	-	249	-	249
Mashonaland West	Zvimba	Chomutamba	3	247	-	-	-	-	247
Masvingo	Chiredzi	Mareya	12	220	218	218	218	218	200
Masvingo	Gutu	Mutendeure	3	192	-	-	-	-	192
Masvingo	Mwenezi	Rudzongwe	12	267	267	267	267	267	210
Matabeleland North	Binga	Siabuwa	3	233	-	-	-	-	233
Matabeleland North	Nkayi	Gonye	12	242	242	211	242	242	193
Matabeleland North	Insiza	Insiza	3	248	-	-	-	-	240
Midlands	Chirumanzu	Gambiza	3	123	-	-	-	-	123
Midlands	Gokwe North	Chireya	3	192	-	-	-	-	192
Midlands	Mberengwa	Mukwakwe	12	177	179	160	179	179	156
Midlands	Mberengwa	Nhemga	3	250	-	-	-	-	248
Midlands	Mberengwa	Zverenje	3	253	-	-	-	-	253
Midlands	Shurugwi	New Gato	12	186	186	186	186	186	186

Of the 8 provinces, 6 had at least one sentinel site included in every MDA, while 2 provinces were not included in all the surveys. Matabeleland South and Mashonaland West were not surveyed in all MDAs. The national survey showed that Matabeleland South had the lowest schistosomiasis prevalence in the country [2] and it was deemed not cost effective to survey this province after every MDA. Therefore, this province was surveyed at the start of this project in 2012 and at the end in 2017, i.e. pre- and post-MDA 1 and post-MDA 6. Similarly other sites including all those in Mashonaland West were sampled at the start-point (MDA1), mid-point (MDA4) and end-point (MDA6) of the control program. These are detailed in **S1 Fig** and in **Table 1.** In the remaining sentinel sites children were surveyed annually for the duration of the study.

For each MDA, the school children were sampled at two points, prior to treatment (pre-MDA) and 6 weeks after treatment was given (post-MDA). Twelve schools (sentinel sites; Gonye, Siabuwa, Muzarabani, Kafura, Chiguri, Mukwakwe, Negato, Mareya, Ruzongwe, Mbembi, Bandanyenje, Kanyaga) (see **Fig 2B**) were followed up annually. As indicated in the study design in **Fig 1**, these 12 schools were purposely selected to represent the full range of the different levels of baseline schistosomiasis infection prevalence i.e. low, moderate and high.

### Study population

The study recruited children aged 6–15 years old and these children would typically be in grades 3–7 in primary school. The children were randomly selected prior to the start of the study in 2012 and the same children were followed up for the duration of the study i.e. until 2017. The selected children were sampled at each sampling point regardless of whether they had received treatment in the last MDA or not. The treatment records of the children were extracted during the post-MDA visit for each MDA, from the MDA records kept by the school. These records informed the treatment coverage rates. Children leave primary school in Zimbabwe for senior school after grade 7, therefore, children who moved out of grade 7 were followed up if enrolled at a local secondary school to ensure the post treatment follow-up. The majority of children lost to follow-up were those who had completed primary school and moved on to secondary school as well as those who transferred to other distant schools.

### Inclusion criteria

To be included in the study, children had to i) provide all 2 stool samples and 3 urine samples for schistosome parasitology and morbidity determination at pre-treatment and 6 weeks post-treatment survey points, ii) have been offered treatment with PZQ during that year’s MDA.

### Study sample sizes

Our national survey sampled 50 children from each school based on the sample size calculation previously published which gave a total sample size of 15, 818 [2] for the national survey. Briefly, in that national survey calculation using 37% as the assumed mean prevalence of schistosomiasis and the error margin of 0.75%, the number of schools selected per district was determined by dividing the district sample size calculated proportionally from the national sample size by the number of children that would be screened per school (n = 50). In this survey, we based our study design on that original study design for consistency and our sample sizes were informed by the original countrywide survey. The present study was to follow the children for 6 years, we were cognizant of the possibility of loss due to migration. Thus, using the national sample size calculation we aimed to recruit a sample size of 250 children per school, with a minimum school sample size of 150 to give a total of 8750 children which would be 55% of the number of children sampled in the national survey. At baseline, a total of 8015 children satisfied the eligibility criteria. Of these children, 7529 (94%) were followed up at the last survey after MDA 6. Of the children from the 12 sentinel sites followed every year, 2781 children were recruited, with 2306 (83%) followed up. Annual sample sizes are summarised in Table 1. Overall there was a loss to follow-up of 6% from MDA1 to MDA6.

### Parasitology

Stool and urine samples were collected from each child between 1000 and 1400 hours, the period when peak egg excretion is expected [16], *S*. *mansoni* infection status and intensity were determined by microscopic examination of slides prepared using the Kato-Katz technique [17] and the formol-ether concentration technique [18] for collecting parasite eggs from stool samples. Two stool samples were collected from each individual over two consecutive days. A single slide was prepared following the Kato-Katz procedure using the 41.7mg template from each stool sample. The egg count from the 41.7mg template was multiplied by 24 as per WHO protocol guidelines (https://www.who.int/medical_devices/diagnostics/selection_in-vitro/selection_in-vitro-meetings/00054_01_kato-katzBench_aids.pdf). The participant’s egg count was the arithmetic mean calculated from the two slides and recoded as the mean eggs per gram of stool. Stool left over from one stool sample was used for the formol-ether concentration technique [18]. There were no indicators for *S*. *mansoni* morbidity assessed due to lack of a point of care rapid diagnostic.

For *S*. *haematobium* infection status and intensity, a urine sample was collected from each participant on three consecutive days. 10mls of this urine was processed following the urine filtration method [19]. Thus, from each participant the three slides were prepared one from each day. The individual’s egg count was the arithmetic mean calculated from the three slides. *S*. *haematobium* morbidity was determined by detection of blood in urine using dipsticks, presence of blood in urine in *S*. *haematobium* endemic areas is also used as indicative of *S*. *haematobium* infection [20]. Individuals were only considered positive if schistosome eggs were detected in their urine/stool samples.

### Antihelminthic treatment

As previously indicated, the treatment was administered by the National Helminth Control treatment team as part of the annual national helminth MDA program. Children were measured using the PZQ dose poles to determine treatment dose and regardless of infection status, were co-administered a standard dose of PZQ i.e. 40mg/kg and albendazole at 400mg per child. During treatment, children were checked to confirm they had swallowed the tablet, and were given bread and juice. Treatment was administered by nurses and the school health coordinators, as is the Ministry of Health practice in Zimbabwe for the national helminth control program. The number and type of tablets administered was recorded for each child in MDA registers, for accurate recording of the MDA reach as well as to distinguish non-compliance from treatment failure in subsequent follow-ups.

### Treatment coverage data

Summary treatment coverage data were available at program level for the country’s MDA program annually for the duration of the study. In 2017, these data were present at district level and the findings of the study were related to these data. All children followed up in the study were confirmed to have received PZQ treatment at each MDA.

### Statistical analysis

Data recorded on field sheets was double entered and proof read in an Excel spreadsheet and comprised data from MDA 1 to 6 with both pre-MDA and post-MDA data recorded for each individual. Infection prevalence and intensity for each time point was calculated for each district.

To determine the effect of treatment on infection level, the ERR, a measure of the change in parasite egg burden upon treatment, and the CR, a measure of those cured of infection upon treatment, were calculated using data from treated children (as confirmed by the school MDA registers) who were positive for schistosome infection using Eqs 1–4 below.

$$Infection\;intensity=\frac{Arithmetic\;Mean\;egg\;count\;for\;all\;individuals}{Total\;number\;of\;individuals\;sampled}$$ Eq 1

$$Prevalence\;=\;\left(\frac{Number\;of\;individuals\;positive\;for\;infection}{Total\;number\;of\;individuals\;sampled}\right)x\;100$$ Eq 2

$$ERR\;(\%)\;=\;\left(1-(\frac{Arithemtic\;mean\;infection\;intensity\;following\;Rx\;}{Arithmetic\;mean\;infection\;intesity\;in\;infected\;people\;at\;baseline})\right)x100$$ Eq 3

$$CR\;(\%)\;=\;\left(\frac{\mathrm{Number}\;of\;individuals\;positive\;at\;baseline\;and\;negative\;following\;R_{X}}{No\;of\;individuals\;positive\;at\;baseline}\right)x100$$ Eq 4

where: ERR = egg reduction rate, CR = cure rate, R_X_ = treatment.

Prevalence data and infection intensities were analysed using Minitab Statistical Software 18 and GraphPad Prism 8. Due to the non-parametric nature of the data, differences in prevalence between annual MDAs were tested for significance using a Fisher’s exact test, whereas difference between pre- and post-MDA time points were analysed using a paired McNemar’s test [21]. Infection intensities between annual MDAs were tested using an unpaired Student’s t-test following Log_10_ (X+1) transformation to normalise data, and between pre- and post-MDA a paired two-way Student’s t-test was calculated. For all analyses, a value of p<0.05 was considered significant. Infection prevalence maps were generated using this primary raw data and plotted using the software package ArcMap 10.1.

## Results

### Effect of treatment in the school children cohort at national level

**Fig 3:**
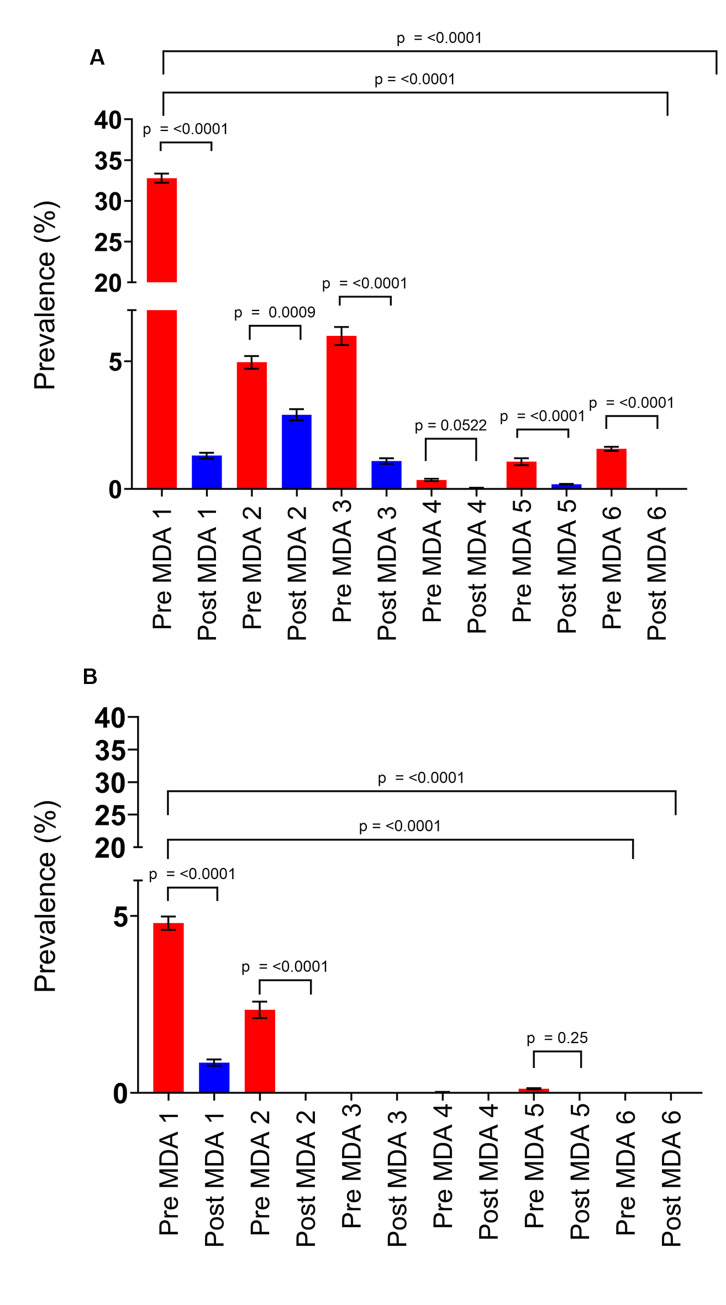
Decline in overall infection prevalence in the cohort of children during the MDAs. Red bars = pre-treatment infection prevalence for each MDA. Blue bars = post-treatment infection prevalence for each MDA. **A**. *S*. *haematobium*. **B**. *S*. *mansoni* as diagnosed through the Kato-Katz procedure during the MDAs. Red bars = pre treatment infection prevalence for each MDA.

Prior to the MDA, *Schistosoma haematobium* prevalence in the sentinel sites was 31.7% and, consistent with Zimbabwe’s 2010 national schistosomiasis survey. *S*. *haematobium* was the most prevalent schistosome species in the country [2]. Following 6 annual rounds of MDA, the prevalence of *S*. *haematobium* decreased significantly to 0% (p<0.0001) (Fig 3A). When comparing infection prevalence pre-treatment, the pre-MDA 1 prevalence of 31.7% decreased to 1.56% (p<0.001) at pre-MDA 6 i.e. 12 months after the last MDA allowing for a full reinfection cycle (Fig 3A).

*Schistosoma mansoni* was tested for using both the Kato-Katz and formol-ether technique, and the prevalence resulting from each at baseline was found to be 4.6% and 1.4% respectively. The results of the more widely used Kato-Katz diagnostic are shown in Fig 3B. After the last MDA in 2017, *S*. *mansoni* prevalence was 0% when tested by both diagnostic techniques (p<0.0001). When comparing infection prevalence pre-treatment, the pre-MDA 1 prevalence of 4.6% by Kato-Katz decreased to 0% (p<0.0001) at pre-MDA 6 and from 1.4% at pre-MDA 1 by formol-ether also to 0% at pre-MDA 6 (see Fig 3B for the Kato-Katz data).

**Fig 4:**
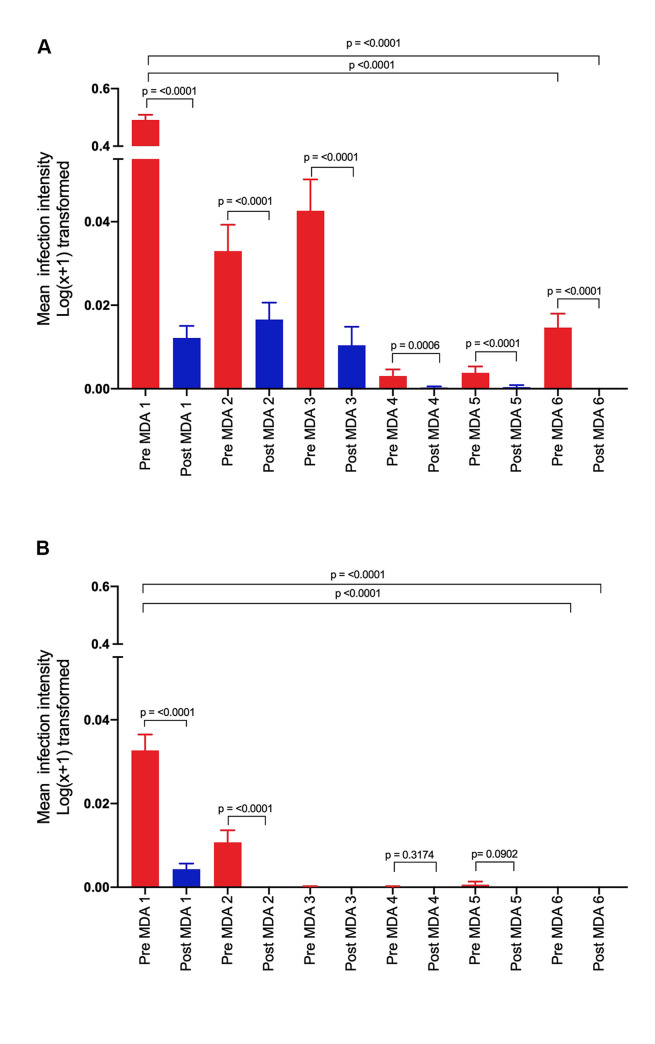
Decline in overall *S*. *haematobium* infection intensity during the MDAs in the cohort of children. Red bars = pre-treatment infection intensity for each MDA. Blue bars = post treatment infection intensity for each MDA**. A.** *S*. *haematobium***, B.** *S*. *mansoni* infection intensity as measured by Kato-Katz.

For both causative species of human schistosomiasis in Zimbabwe, *S*. *haematobium* and *S*. *mansoni*, arithmetic mean infection intensity (analysed using the Log_10_(mean egg count+1) transformation) in the cohort of school children decreased significantly from the initiation of the MDA programme to the end (p = <0.0001) (see Fig 4). *S*. *haematobium* decreased from a mean of 0.49 (SEM = 0.01) to 0.07 (SEM 0.02) at pre-MDA 6 (see Fig 4A) and 0 (SEM = 0) at post-MDA 6. *S*. *mansoni* infection intensity determined by Kato-Katz decreased from 0.03 (SEM = 0.002) at pre-MDA 1 to 0 (SEM = 0) at both pre- and post-MDA 6, and from 0.006 (SEM = 0.0007) at pre-MDA 1 to 0 (SEM = 0) again at both pre- and post-MDA 6 using the formol-ether diagnostic technique (4b).

### Schistosome morbidity

**Fig 5:**
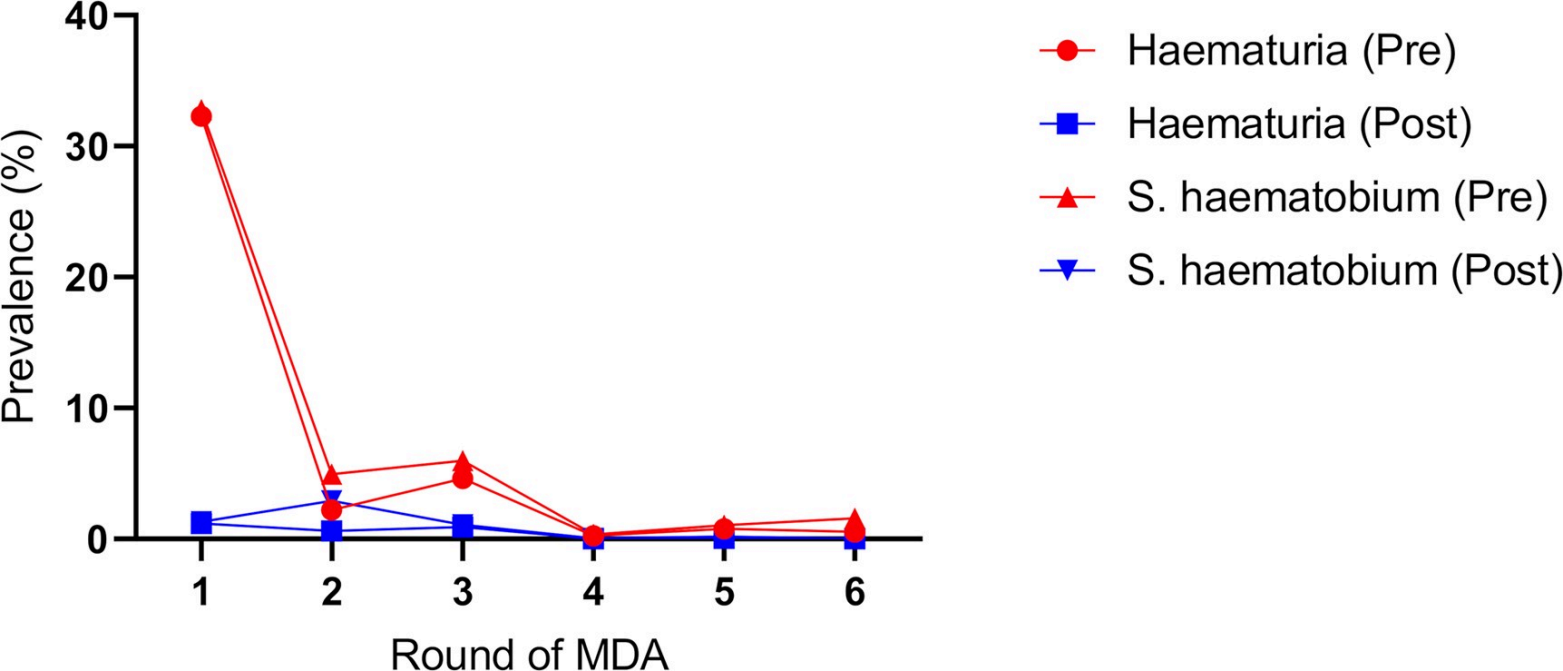
Decline in the prevalence of *S. haematobium* morbidity as measured by visible haematuria in the cohort of children.

Macro or visible haematuria measured as a marker of morbidity of *S*. *haematobium* infection fell significantly from 32.3% in pre-MDA 1 to 0% at post-MDA 6 (p <0.001) and the gradual decline is shown in **Fig 5**. The prevalence of haematuria had fallen to 0% prior to the 6^th^ MDA.

### Effect of treatment in the school cohort at province level

**Fig 6:**
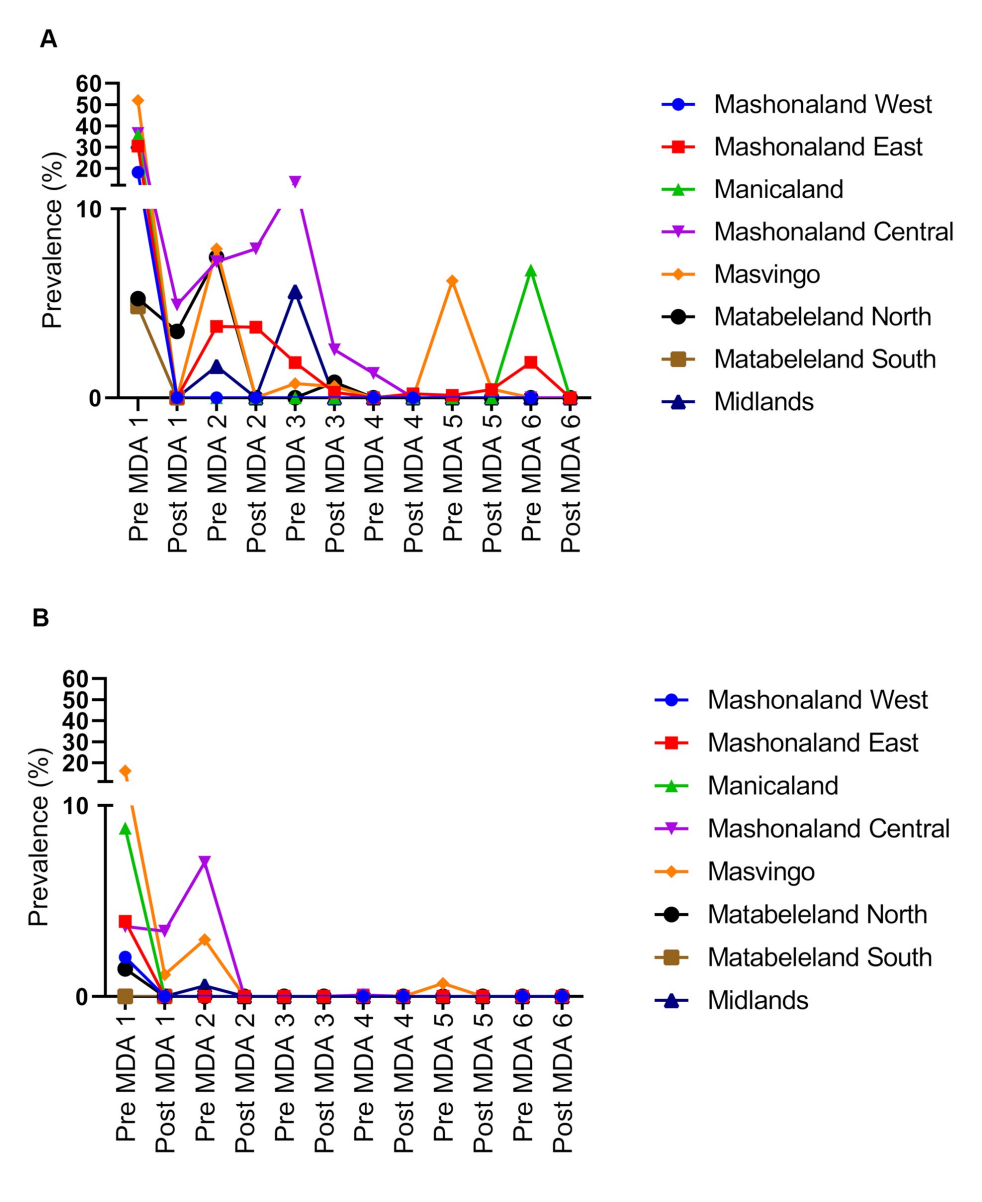
Decline in infection prevalence at province level during the MDAs. **A**. *S*. *haematobium*. **B.** S. *mansoni* as measured by Kato-Katz in the cohort of children.

The impact of treatment was investigated in all 8 provinces. The study shows that the first MDA had a significant effect on infection levels. Investigations in the sentinel sites in the 6 provinces surveyed at every MDA for the 6 MDAs shows a gradual decline in infection level. Fig 6A shows the decline in *S*. *haematobium* infection prevalence while Fig 6B shows the decline in *S*. *mansoni* prevalence as assessed by Kato-Katz.

Matabeleland North started with a prevalence of 5.3% for *S*. *haematobium* and 1.5% for *S*. *mansoni* in 2012 and decreased in both species to 0% by 2017. Masvingo had the highest starting prevalence among the provinces, calculated at 51.9% for *S*. *haematobium* and 16.1% for *S*. *mansoni* in 2012. These both decreased to 0% following the last MDA in 2017. The prevalence of *S*. *haematobium* in the Midlands was 32.3% in 2012 decreasing to 0% in 2017 and S. *mansoni* prevalence in the Midlands started at 0% in 2012, and remained at 0% in 2017. Mashonaland West province had a prevalence of 18.1% for *S*. *haematobium* and 2.1% in *S*. *mansoni* in 2012, both of which decreased to 0% in 2017. Mashonaland Central had a prevalence of 36.4% for *S*. *haematobium* and 3.7% for *S*. *mansoni* in 2012, and again, both decreased to 0% in 2017. Mashonaland East had an initial prevalence of 30.4% for *S*. *haematobium* and 3.9% for *S*. *mansoni*. These decreased to 0% after the 6^th^ MDA. Manicaland province had a prevalence of 35.6% for *S*. *haematobium* and 8.8% for *S*. *mansoni* before the first MDA, and both decreased to 0% after the last MDA. Overall, the final data collected following the 2017 annual MDA revealed that every province had decreased to 0% for both *S*. *haematobium* and *S*. *mansoni*.

The overall change in prevalence between pre-MDA 1 and post-MDA 6 was significant in both *S*. *haematobium* (p = <0.0001) and *S*. *mansoni* (p = <0.0001) (Fig 2). When investigating this at a province level, the difference in prevalence of *S*. *haematobium* was significant for Mashonaland West, East, Central, Midlands, Masvingo, Manicaland (all p = <0.0001) and Matabeleland North (p = 0.0002). The change in prevalence of *S*. *mansoni* on the province level was also significant in Mashonaland East, West, Central, Manicaland and Masvingo (all p = <0.0001), however Matabeleland North was not (p = 0.2000) and Midlands had no detectable *S*. *mansoni* at either time points. The impact of the MDAs on both schistosome species is shown in **S2 Fig**.

### Effect of treatment in the school cohort at district level

Of the 29 districts investigated in MDA 1, 24 (82.8%) districts were positive for *S*. *haematobium*, 33.3% for both and 10 (34.5%) districts were positive for *S*. *mansoni*. District prevalences ranged from 0% to 88.1% pre-MDA 1 for *S*. *haematobium* and 0% to 25.5% for *S*. *mansoni* as shown by the data of pre-MDA 1 prevalences in Fig 6A for *S*. *haematobium* and Fig 6B for *S*. *mansoni*. Infection prevalence in all districts was 0% post-MDA 6 for both species.

**Fig 7:**
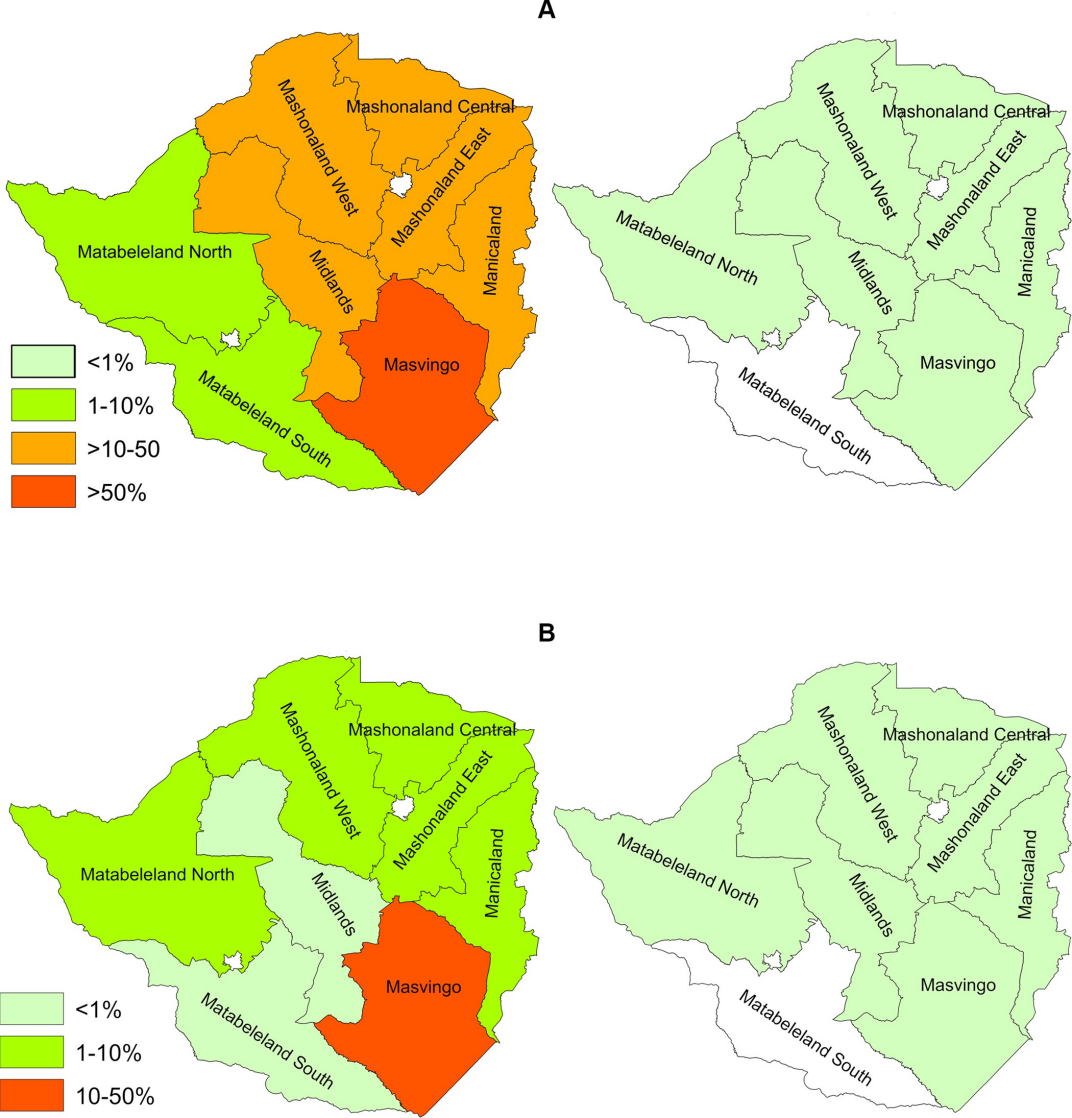
Changes in prevalence at national level in the cohort of children. **A**. *S*. *haematobium*. **B**. *S*. *mansoni*. The maps were generated using GIS raw data for the schools using ArcMap 10.1.

The summary of the overall impact on national prevalence in the cohort of children, presenting the initial data collected prior to the commencement of the first MDA in 2012, to the final measurements taken following the 6^th^ MDA in 2017 is shown in Fig 7.

### Egg reduction and cure rates

The egg reduction rates (ERR) and cure rates (CR) were calculated for each MDA. Overall ERR and CR are given in Tables 2 and 3 respectively. Across all provinces, for both *S*. *mansoni* and *S*. *haematobium*, ERR were all above 90%. Across the MDAs, average CR ranged from 83.4% to 100% for *S*. *haematobium* and 92.8% to 100% for *S*. *mansoni* diagnosed by Kato-Katz and 95.8% to 100% for *S*. *mansoni* diagnosed by formol-ether technique.

**Table 2:** Mean egg reduction rate (%).

MDA	*S*. *haematobium*	*S*. *mansoni* (Kato-Katz)	*S*. *mansoni* (Formol-Ether)
1	99.29	95.72	98.58
2	91.16	N/A	100
3	96.99	100	N/A
4	100	100	N/A
5	97.77	100	N/A
6	100	N/A	N/A

N/A represents MDA where the pre-treatment infection intensity was 0 eggs/ml or 0 eggs/mg.

**Table 3:** Mean cure rates (%).

MDA	*S*. *haematobium*	*S*. *mansoni (Kato-Katz)*	*S*. *mansoni* (Formol-Ether)
1	93.98	92.83	95.83
2	84.84	100.00	100.00
3	83.39	N/A	N/A
4	100.00	100.00	N/A
5	100.00	100.00	100.00
6	100.00	N/A	N/A

N/A represents MDA where the pre-treatment infection intensity was 0 eggs/ml or 0 eggs/mg.

### Treatment coverage

**Fig 8:**
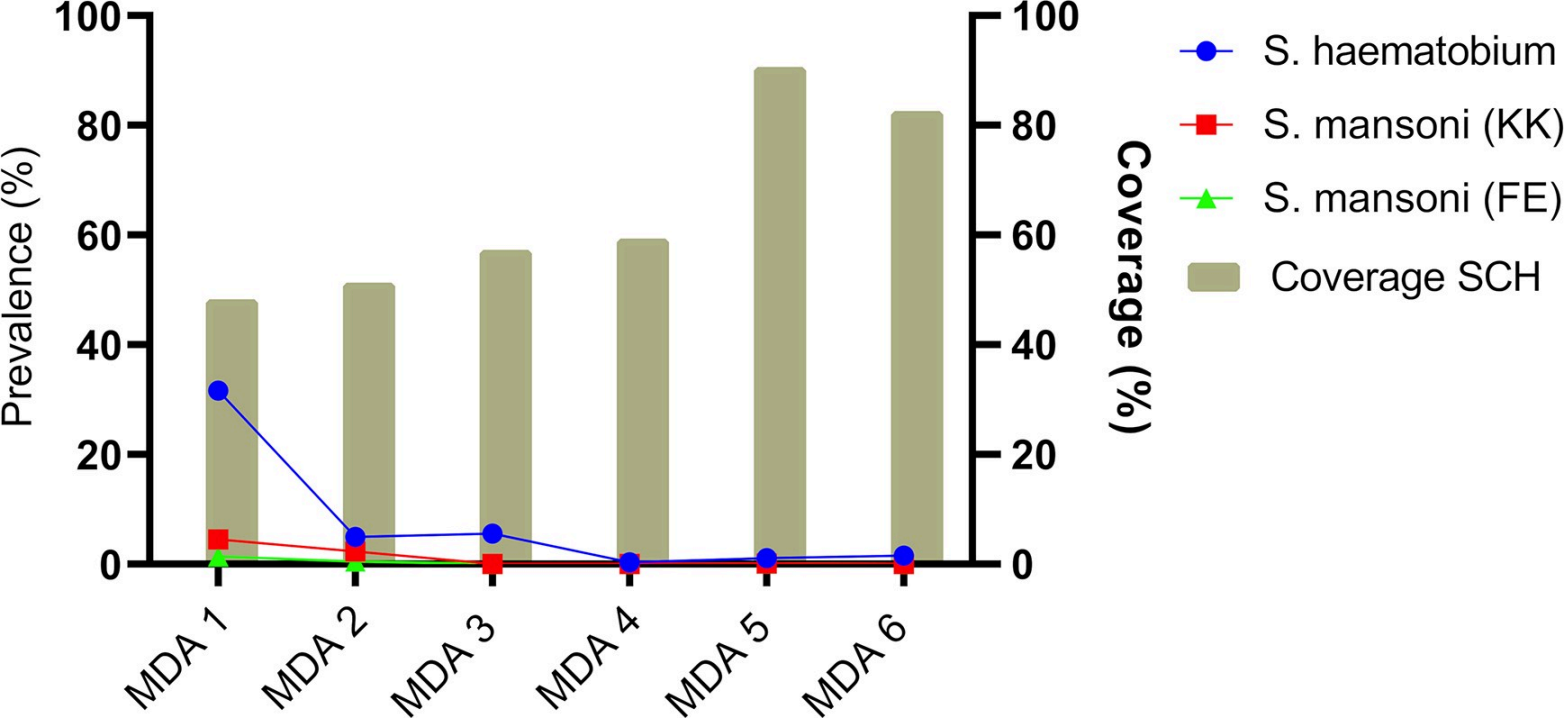
Relationship between treatment coverage rates and schistosome infection prevalence across the MDAs in the cohort of children.

Mean coverage across all the provinces was lowest in MDA 1 at 48%, but this increased gradually peaking at 90.3% in the cohort during MDA 5 (Fig 8). While the mean national coverage at MDA 6 was 82.3% there was heterogeneity between districts with coverage ranging from 73.5% (Mashonaland West) to 96.5% (Mashonaland Central) for MDA 6 (**S3 Fig**).

## Discussion

The Uniting to Combat NTDs score card calculates Zimbabwe’s efforts to combat NTDs in terms of average of coverage across the five diseases amenable to mass treatment (schistosomiasis, soil transmitted helminths (STH), river blindness, trachoma, elephantitis) as 12% but indicates that the coverage for schistosomiasis is 100% (see https://unitingtocombatntds.org/africa/zimbabwe/). However, these score card data do not indicate the effectiveness of the country’s helminth control program in terms of reducing infection level or morbidity. This study presents the results from the analysis of the effect of annual praziquantel treatment on schistosome infection and morbidity in a cohort of primary school children during the national helminth control program in Zimbabwe. The study showed that upon sustained annual MDA using PZQ to treat schistosome infections and morbidity, both schistosome infection and morbidity have been significantly reduced in this cohort of children, meeting the WHO aim of reducing morbidity and infection in school-aged children [22].

Zimbabwe adopted a country strategy to treat all affected areas annually for 6 years. By surveying sentinel sites before the MDA and 6 weeks after the MDA every year, we have been able to demonstrate that following the third MDA, the prevalence of schistosomiasis in the cohort of school children in every province fell under the low-risk category deemed by the WHO i.e. prevalence of less than 10% [14][15] and remained there consistently throughout the rest of the programme.

Zimbabwe is one of the 41 African countries mentioned in the latest WHO Report on NTDs [23], as currently having preventive chemotherapy programs for schistosomiasis. Of these countries 28 (68.3%) which include Zimbabwe, were implementing preventative chemotherapy programs in 2015. Of these only 15 had extended coverage to all endemic areas. In addition to this annual treatment across all levels of schistosome endemicity, the treatment coverage obtained each year generally increased from MDA 1 throughout the program. Maximum coverage reached for all school children targeted in Zimbabwe’s helminth control program which included the cohort of children in this study, was 90%. This was above the recommended 75% coverage of school-aged children as detailed in the “2020 roadmap” set out by the WHO [9].

While there was an overall reduction in infection prevalence and intensity over the duration of the control program in the cohort of school children, there was some rebound in infection particularly over the first 3 years. This is unsurprising as there were no control efforts directed at breaking the transmission cycle. Studies in Kenya indicate that the reinfection can be sufficiently intense as to warrant more than an annual treatment [24]. In Zimbabwe, with increasing treatment coverage came lower re-infection rates culminating in the reduction of prevalence to 0% in this cohort of school children following the last MDA.

For both *S*. *mansoni* and *S*. *haematobium*, ERR were above 90%. Across the MDAs, average CR ranged from 83.4% to 100% for *S*. *haematobium* and 92.8% to 100% for *S*. *mansoni* diagnosed by Kato-Katz and 95.8% to 100% for *S*. *mansoni* diagnosed by formol-ether technique. Variations in treatment outcome have been previously reported and have prompted studies into trying to identify better measures of PZQ efficacy since parasite egg excretion is only a proxy for drug effects on adult worms [25]. Egg excretion can be confounded by various host or parasite factors, including facultative temporary cessation of excretion by the adult worms [25]. Variation in both CR and ERR measures could be attributable to parasite- or host-based factors including varying efficacy of PZQ in the killing of *S*. *haematobium* and *S*. *mansoni* in co-endemic areas [26], the intensity of the initial infection, host genetics, and the sensitivity of the diagnostic tests used to identify either species. For example, the urine filtration technique and Kato-Katz technique can miss low-level infections [27]. In areas with a particularly high prevalence of schistosomiasis, there is a greater chance of individuals being infected with immature schistosomes at the time of MDA treatment and therefore will not be completely cleared by one dose of praziquantel [28]. Furthermore, in areas of high transmission, high levels of reinfection can occur especially if the MDA occurs before the transmission period. Treatment of only school children excluding adults and preschool children potentially maintains transmission. All of these factors may explain the observed persistence of infection post-treatment, such as that of Mashonaland Central in both *Schistosoma* species, for the first 3 MDAs in this study [29, 30].

The possibility of the parasites developing resistance to PZQ is always a present threat, with reports of low CR in certain regions [31, 32]. Indeed reduced efficacy of PZQ has been reported following multiple rounds of MDA in Uganda for *S*. *mansoni* [33]. To counteract these fears, investigations into multiple cases of laboratory and field PZQ resistance determined that the reduced efficacy of drug treatment due to resistance was rare [34]. Furthermore, a meta-analysis of PZQ CR and ERR we have conducted indicates that these have not changed significantly in the past 20 years. The reduction of infection at the sentinel sites to 0% and 0 egg counts for both schistosome species reflects that treatment remained efficacious throughout the MDA period. As Zimbabwe intensifies control efforts to move towards eliminating schistosomiasis as a public health problem, it will be important to collect parasite samples from infection hot spots to determine causes of persistent infection [35].

While Zimbabwe has made significant progress towards the control of schistosomiasis through this 6-year MDA program, more still needs to be done; first to maintain the low infections achieved in the primary school children who were the target of Zimbabwe’s helminth control program represented by the cohort of children studied here and, second to include all populations at risk of infection i.e. adults and preschool children. The 2012 World Health Assembly resolution 65.21 advocates for elimination of schistosomiasis transmission in member states. This is an aspirational goal for Zimbabwe although in practise a difficult task. Annual MDA is not sustainable long-term for most endemic countries both for economic and logistical reasons in health systems with low budgets. It is also not effective on its own, at meeting the elimination aspiration of WHA 65.21. Thus, Zimbabwe, similar to all other schistosome endemic countries aiming to eliminate the disease, needs to strengthen concerted efforts to both reduce infection and morbidity, and also interrupt transmission. This must be based on integrated control approaches including intense PZQ treatment targeted at any hot spots of infection/transmission, snail control, treatment of infective water and improved access to safe water, sanitation and hygiene (WASH) [36]. The impact of such an integrated approach has been demonstrated in Egypt, where schistosomiasis is being tackled through targeted MDA and WASH improvements [37]. The lack of integrated comprehensive approaches to reduce both infection levels and transmission as well as the short-term timescale of MDA may explain the challenge experienced by other counties for example, Kenya [24] and Uganda [38], in improving the impact of MDA alone on infection levels/transmission. A comprehensive national impact assessment exercise for the national control program as is planned by Zimbabwe’s Ministry of Health at the end of this initial phase of the country’s helminth control program will be informative in shaping the next stages in the country’s control of schistosomiasis.

We designed the study sampling framework based on the results of the national control survey which formed the basis of Zimbabwe’s national control program [2]. Surveying all sentinel sites at every survey point would have given more information, but the perceived benefit from this did not justify the associated expense. In the national survey, 50 children per school were sampled, whereas in this study, in the majority of schools we sampled at least 200 children. Sample sizes lower than 200 were reflective of the enrolment numbers of children of the appropriate age group at the school. The follow-up of 75% of the children over the 6 years reflects predominantly children who moved school to a different area and could not be traced. Children remained eligible for follow-up if they moved to the local catchment area senior school i.e. within the same district and often served by the same river system to avoid following children who moved to areas of different schistosome transmission dynamics.

The data were presented summarily at province level; however, as control efforts strengthen in the move towards elimination, more refined mapping at district level, therefore more schools, will be more informative. For infection diagnosis, we used the parasitological examination of urine and stool samples and this showed the reduction of egg counts to 0 for both species of schistosome infections. Although the parasitological examination of urine and stool is the currently recommend diagnostic for schistosomiasis, we and others have previously shown that the approach is less sensitive when infection levels are low [27] and therefore more accurate diagnostic tools are needed to monitor progress of control efforts. Alternative diagnostic approaches including PCR [39] and parasite antigens [40–43] have been used by others, albeit not routinely in national MDA programs where there may be challenges with diagnostic accuracy [44]. Although beyond the scope of this study, information on the transmission dynamics in the local infective water bodies which the children frequented would have added valuable information on the impact of treatment on the force of infection.

On a schistosome elimination agenda, there is need to extend treatment to cover the whole community, including preschool aged children (aged 5 years and below) as well as adults. Indeed the guidelines from WHO indicate that preventative chemotherapy should be targeted at all groups at risk of infection in highly endemic communities [45]. Quantitative work has shown that integrated community-wide treatment for schistosomiasis and soil-transmitted helminths can be highly cost effective even in areas of low endemicity for either schistosomiasis or STH [46]. It is also important to be cognizant of the fact the current WHO guidelines were not designed with the goal of elimination so current control strategies will need to be adapted to deliver elimination while new or better tools for diagnosis and verification of cessation of transmission will need to be developed. Indeed, the WHO already acknowledges the need for flexibility in control approaches for different counties in the roadmap for controlling and eliminating NTDs [9]. Thus Zimbabwe’s approach of annual treatments regardless of the schistosomiasis endemicity level at the operational region may have been more effective. Zimbabwe’s last MDA was in 2017 and the country is now evaluating the impact of the control program to inform the next steps in the country’s helminth control action plan.

In conclusion, based on the survey of schistosome infection and *S*. *haematobium* morbidity in a cohort of school children in sentinel sites across the country, Zimbabwe’s helminth control program significantly reduced schistosome infection and morbidity levels in this cohort of school-age children to levels where elimination is a possible goal. If extrapolated to national level, the control program moved the country from a moderate to low endemicity levels as per WHO classification for schistosomiasis [14]. The findings of this study will inform the design of the country’s integrated control strategy towards elimination following a national assessment of the impact of the national helminth control program at all previously surveyed sites.

## Supporting information

S1 Fig The 35 sentinel sites chosen to represent districts (and thus provinces) in the country. The districts are colour coded to represent baseline schistosome infection prevalence classified into low infection (coloured grey), moderate infection (coloured blue) and heavy infection (coloured red) and indicating the number of surveys conducted per sentinel site. Mashonaland and Matabeleland Provinces are abbreviated thus: Mash West = Mashonaland West, Mash East = Mashonaland East, Mash C = Mashonaland Central, Mat N = Matabeleland North, Mat S = Matabeleland South.
(DOCX)

S2 Fig Combined prevalence of *S*. *haematobium* and *S*. *mansoni* in the cohort of Zimbabwean school children (a) before year 2012, and (b) after year 2017 Mass Drug Administration with Praziquantel. Maps were generated using the primary raw data and plotted using ArcMap 10.1.
(PDF)

S3 Fig Summary % coverage of schistosome treatment at provincial level in the school children cohort in the last MDA in 2017. (TIF)
