# Supplementary material for: Six rounds of annual praziquantel treatment during a national helminth control program significantly reduced schistosome infection and morbidity levels in a cohort of schoolchildren in Zimbabwe
Source: PLoS Negl Trop Dis. 2020 Jun 22;14(6):e0008388. doi: 10.1371/journal.pntd.0008388 (PMC7332090; doi:10.1371/journal.pntd.0008388)
Supplement: S1 Fig — The districts are colour coded to represent baseline schistosome infection prevalence classified into low infection (coloured grey), moderate infection (coloured blue) and heavy infection (coloured red) and indicating the number of surveys conducted per sentinel site. Mashonaland and Matabeleland Provinces are abbreviated thus: Mash West = Mashonaland West, Mash East = Mashonaland East, Mash C = Mashonaland Central, Mat N = Matabeleland North, Mat S = Matabeleland South. (DOCX) [file pntd.0008388.s001.docx]

| **Province** | **District**  **baseline prevalence indicated by colour coding** |  | **sampled** | **sampled** |
| --- | --- | --- | --- | --- |
| **Mash West** | **Chegutu** |  | **Pre** | **Post** |
|  |  | **MDA 1** | Y | Y |
|  |  | **MDA 2** | . | . |
|  |  | **MDA 3** | . | . |
|  |  | **MDA 4** | Y | Y |
|  |  | **MDA 5** | . | . |
|  |  | **MDA 6** | Y | Y |
|  | **Hurungwe** |  | **Pre** | **Post** |
|  |  | **MDA 1** | Y | Y |
|  |  | **MDA 2** | . | . |
|  |  | **MDA 3** | . | . |
|  |  | **MDA 4** | Y | Y |
|  |  | **MDA 5** | . | . |
|  |  | **MDA 6** | Y | Y |
|  | **Makonde** |  | **Pre** | **Post** |
|  |  | **MDA 1** | Y | Y |
|  |  | **MDA 2** | . | . |
|  |  | **MDA 3** | . | . |
|  |  | **MDA 4** | Y | Y |
|  |  | **MDA 5** | . | . |
|  |  | **MDA 6** | Y | Y |
|  | **Zvimba** |  | **Pre** | **Post** |
|  |  | **MDA 1** | Y | Y |
|  |  | **MDA 2** | . | . |
|  |  | **MDA 3** | . | . |
|  |  | **MDA 4** | . | . |
|  |  | **MDA 5** | . | . |
|  |  | **MDA 6** | N | Y |
| **Mash East** | **Chikomba** |  | **Pre** | **Post** |
|  |  | **MDA 1** | Y | Y |
|  |  | **MDA 2** | . | . |
|  |  | **MDA 3** | . | . |
|  |  | **MDA 4** | Y | Y |
|  |  | **MDA 5** | . | . |
|  |  | **MDA 6** | Y | Y |
|  | **Murehwa** |  | **Pre** | **Post** |
|  |  | **MDA 1** | Y | Y |
|  |  | **MDA 2** | Y | Y |
|  |  | **MDA 3** | Y | Y |
|  |  | **MDA 4** | Y | Y |
|  |  | **MDA 5** | Y | Y |
|  |  | **MDA 6** | Y | Y |
|  | **Mutoko** |  | **Pre** | **Post** |
|  |  | **MDA 1** | Y | Y |
|  |  | **MDA 2** | Y | Y |
|  |  | **MDA 3** | Y | Y |
|  |  | **MDA 4** | Y | Y |
|  |  | **MDA 5** | Y | Y |
|  |  | **MDA 6** | Y | Y |
|  | **UMP** |  | **Pre** | **Post** |
|  |  | **MDA 1** | Y | Y |
|  |  | **MDA 2** | Y | Y |
|  |  | **MDA 3** | Y | Y |
|  |  | **MDA 4** | Y | Y |
|  |  | **MDA 5** | Y | Y |
|  |  | **MDA 6** | Y | Y |
| **Manicaland** | **Makoni** |  | **Pre** | **Post** |
|  |  | **MDA 1** | Y | Y |
|  |  | **MDA 2** | Y | Y |
|  |  | **MDA 3** | Y | Y |
|  |  | **MDA 4** | Y | Y |
|  |  | **MDA 5** | Y | Y |
|  |  | **MDA 6** | Y | Y |
|  | **Buhera** |  | **Pre** | **Post** |
|  |  | **MDA 1** | Y | Y |
|  |  | **MDA 2** | . | . |
|  |  | **MDA 3** | . | . |
|  |  | **MDA 4** | Y | Y |
|  |  | **MDA 5** | . | . |
|  |  | **MDA 6** | Y | Y |
|  | **Chipinge** |  | **Pre** | **Post** |
|  |  | **MDA 1** | Y | Y |
|  |  | **MDA 2** | . | . |
|  |  | **MDA 3** | . | . |
|  |  | **MDA 4** | Y | Y |
|  |  | **MDA 5** | . | . |
|  |  | **MDA 6** | Y | Y |
|  | **Bikita** |  | **Pre** | **Post** |
|  |  | **MDA 1** | Y | N |
|  |  | **MDA 2** | . | . |
|  |  | **MDA 3** | . | . |
|  |  | **MDA 4** | . | . |
|  |  | **MDA 5** | . | . |
|  |  | **MDA 6** | N | Y |
|  | **Nyanga** |  | **Pre** | **Post** |
|  |  | **MDA 1** | Y | Y |
|  |  | **MDA 2** | . | . |
|  |  | **MDA 3** | . | . |
|  |  | **MDA 4** | Y | Y |
|  |  | **MDA 5** | . | . |
|  |  | **MDA 6** | Y | Y |
| **Mash C** | **Muzarabani** |  | **Pre** | **Post** |
|  |  | **MDA 1** | Y | Y |
|  |  | **MDA 2** | Y | Y |
|  |  | **MDA 3** | Y | Y |
|  |  | **MDA 4** | Y | Y |
|  |  | **MDA 5** | Y | Y |
|  |  | **MDA 6** | Y | Y |
|  | **Mt Darwin** |  | **Pre** | **Post** |
|  |  | **MDA 1** | Y | Y |
|  |  | **MDA 2** | Y | Y |
|  |  | **MDA 3** | Y | Y |
|  |  | **MDA 4** | Y | Y |
|  |  | **MDA 5** | Y | Y |
|  |  | **MDA 6** | Y | Y |
|  | **Rushinga** |  | **Pre** | **Post** |
|  |  | **MDA 1** | Y | Y |
|  |  | **MDA 2** | Y | Y |
|  |  | **MDA 3** | Y | Y |
|  |  | **MDA 4** | Y | Y |
|  |  | **MDA 5** | Y | Y |
|  |  | **MDA 6** | Y | Y |
|  | **Guruve** |  | **Pre** | **Post** |
|  |  | **MDA 1** | Y | Y |
|  |  | **MDA 2** | . | . |
|  |  | **MDA 3** | . | . |
|  |  | **MDA 4** | Y | Y |
|  |  | **MDA 5** | . | . |
|  |  | **MDA 6** | Y | Y |
|  | **Mazowe** |  | **Pre** | **Post** |
|  |  | **MDA 1** | Y | Y |
|  |  | **MDA 2** | . | . |
|  |  | **MDA 3** | . | . |
|  |  | **MDA 4** | Y | Y |
|  |  | **MDA 5** | . | . |
|  |  | **MDA 6** | Y | Y |
|  | **Shamva** |  | **Pre** | **Post** |
|  |  | **MDA 1** | Y | Y |
|  |  | **MDA 2** | . | . |
|  |  | **MDA 3** | . | . |
|  |  | **MDA 4** | Y | Y |
|  |  | **MDA 5** | . | . |
|  |  | **MDA 6** | Y | Y |
| **Masvingo** | **Chiredzi** |  | **Pre** | **Post** |
|  |  | **MDA 1** | Y | Y |
|  |  | **MDA 2** | Y | Y |
|  |  | **MDA 3** | Y | Y |
|  |  | **MDA 4** | Y | Y |
|  |  | **MDA 5** | Y | Y |
|  |  | **MDA 6** | Y | Y |
|  | **Gutu** |  | **Pre** | **Post** |
|  |  | **MDA 1** | Y | Y |
|  |  | **MDA 2** | . | . |
|  |  | **MDA 3** | . | . |
|  |  | **MDA 4** | . | . |
|  |  | **MDA 5** | . | . |
|  |  | **MDA 6** | N | Y |
|  | **Mwenezi** |  | **Pre** | **Post** |
|  |  | **MDA 1** | Y | Y |
|  |  | **MDA 2** | Y | Y |
|  |  | **MDA 3** | Y | Y |
|  |  | **MDA 4** | Y | Y |
|  |  | **MDA 5** | Y | Y |
|  |  | **MDA 6** | Y | Y |
| **Mat N** | **Binga** |  | **Pre** | **Post** |
|  |  | **MDA 1** | Y | Y |
|  |  | **MDA 2** | . | . |
|  |  | **MDA 3** | . | . |
|  |  | **MDA 4** | . | . |
|  |  | **MDA 5** | . | . |
|  |  | **MDA 6** | N | Y |
|  | **Nkayi** |  | **Pre** | **Post** |
|  |  | **MDA 1** | Y | Y |
|  |  | **MDA 2** | Y | Y |
|  |  | **MDA 3** | Y | Y |
|  |  | **MDA 4** | Y | Y |
|  |  | **MDA 5** | Y | Y |
|  |  | **MDA 6** | Y | Y |
| **Mat S** | **Insiza** |  | **Pre** | **Post** |
|  |  | **MDA 1** | Y | Y |
|  |  | **MDA 2** | . | . |
|  |  | **MDA 3** | . | . |
|  |  | **MDA 4** | . | . |
|  |  | **MDA 5** | . | . |
|  |  | **MDA 6** | N | Y |
| **Midlands** | **Chirumanzu** |  | **Pre** | **Post** |
|  |  | **MDA 1** | Y | Y |
|  |  | **MDA 2** | . | . |
|  |  | **MDA 3** | . | . |
|  |  | **MDA 4** | . | . |
|  |  | **MDA 5** | . | . |
|  |  | **MDA 6** | N | Y |
|  | **Gokwe North** |  | **Pre** | **Post** |
|  |  | **MDA 1** | Y | Y |
|  |  | **MDA 2** | . | . |
|  |  | **MDA 3** | . | . |
|  |  | **MDA 4** | . | . |
|  |  | **MDA 5** | . | . |
|  |  | **MDA 6** | N | Y |
|  | **Mberengwa** |  | **Pre** | **Post** |
|  |  | **MDA 1** | Y | Y |
|  |  | **MDA 2** | Y | Y |
|  |  | **MDA 3** | Y | Y |
|  |  | **MDA 4** | Y | Y |
|  |  | **MDA 5** | Y | Y |
|  |  | **MDA 6** | Y | Y |
|  | **Shurugwi** |  | **Pre** | **Post** |
|  |  | **MDA 1** | Y | Y |
|  |  | **MDA 2** | Y | Y |
|  |  | **MDA 3** | Y | Y |
|  |  | **MDA 4** | Y | Y |
|  |  | **MDA 5** | Y | Y |
|  |  | **MDA 6** | Y | Y |
