# Supplementary figures and images for: Six rounds of annual praziquantel treatment during a national helminth control program significantly reduced schistosome infection and morbidity levels in a cohort of schoolchildren in Zimbabwe
Source: PLoS Negl Trop Dis. 2020 Jun 22;14(6):e0008388. doi: 10.1371/journal.pntd.0008388 (PMC7332090; doi:10.1371/journal.pntd.0008388)

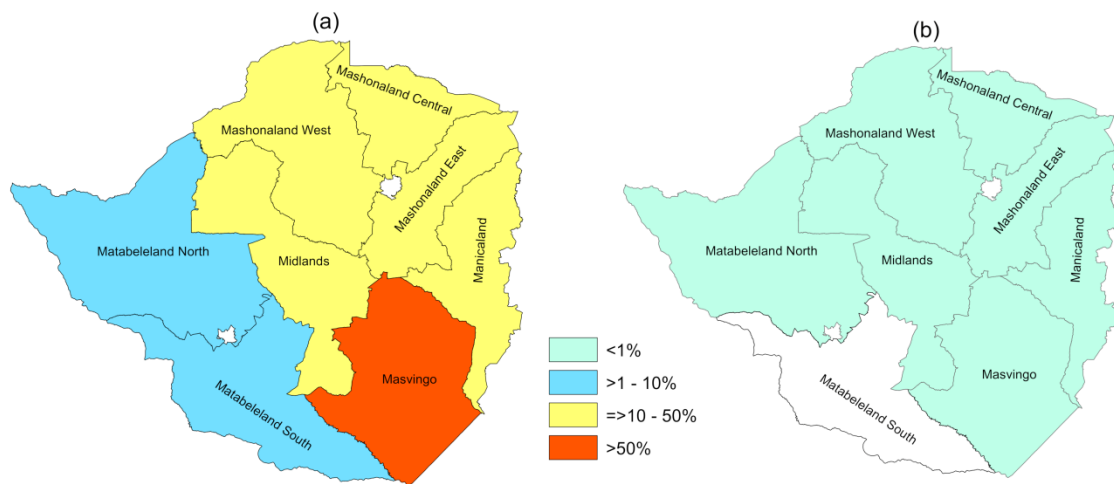

Supplement: S2 Fig — Combined prevalence of S. haematobium and S. mansoni in the cohort of Zimbabwean school children (a) before year 2012, and (b) after year 2017 Mass Drug Administration with Praziquantel. Maps were generated using the primary raw data and plotted using ArcMap 10.1. (PDF) [file pntd.0008388.s002.pdf]

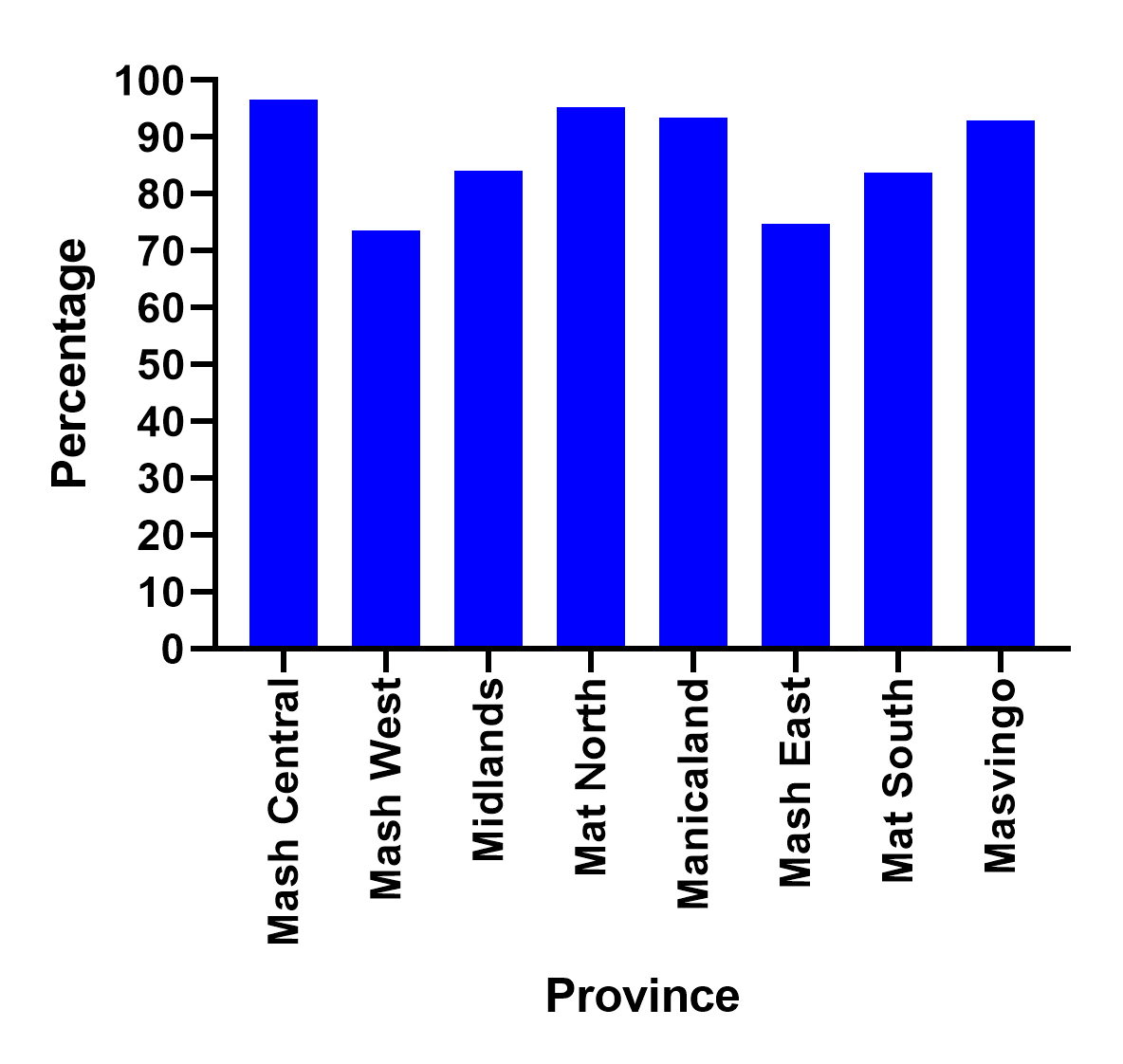

Supplement: S3 Fig — (TIF) [file pntd.0008388.s003.tif]
